# From Processivity to Genome Maintenance: The Many Roles of Sliding Clamps

**DOI:** 10.3390/genes13112058

**Published:** 2022-11-07

**Authors:** Meenakshi Mulye, Manika Indrajit Singh, Vikas Jain

**Affiliations:** Microbiology and Molecular Biology Laboratory, Department of Biological Sciences, Indian Institute of Science Education and Research (IISER), Bhopal 462066, India

**Keywords:** sliding clamp, processivity factor, dimer, trimer, moonlight role

## Abstract

Sliding clamps play a pivotal role in the process of replication by increasing the processivity of the replicative polymerase. They also serve as an interacting platform for a plethora of other proteins, which have an important role in other DNA metabolic processes, including DNA repair. In other words, clamps have evolved, as has been correctly referred to, into a mobile “tool-belt” on the DNA, and provide a platform for several proteins that are involved in maintaining genome integrity. Because of the central role played by the sliding clamp in various processes, its study becomes essential and relevant in understanding these processes and exploring the protein as an important drug target. In this review, we provide an updated report on the functioning, interactions, and moonlighting roles of the sliding clamps in various organisms and its utilization as a drug target.

## 1. Introduction

DNA replication is the first key step of the central dogma of molecular biology that ensures the faithful duplication of the entire genome of a cell. The enzyme DNA polymerase (DNAP) required for this process is universally present in all living forms [1,2]. DNAPs are large multi-subunit enzymes that work with other accessory proteins such as helicases, primases, sliding clamps, single-strand DNA-binding proteins, topoisomerases, gyrases, DNA ligases, and telomerases to successfully carry out DNA replication [3,4]. Together, these proteins form a ‘replisome’, and unwind DNA to synthesize complementary strands using both the parent strands as a template in a semi-conservative manner [5]. Smooth execution of the entire process, i.e., initiation, elongation, and termination, requires the proper positioning and functioning of all the proteins. DNAPs display high processivity (carry out rapid catalysis) and high fidelity (less error prone) [1]. To achieve high processivity, DNAPs utilize processivity factors [6,7].

DNAP processivity factors, commonly referred to as sliding clamp proteins, are ring-shaped multi-subunit proteins that encircle DNA and provide processivity to the DNAP primarily by holding it onto DNA and enhancing the rate of nucleotide incorporation up to 100–1000 folds in the growing DNA strand [8,9,10]. Sliding clamps have a far-reaching presence in almost all organisms, including bacteriophage, and are critical for survival because of their role in increasing the processivity of DNA polymerases. They bind and slide freely onto the DNA to regulate various interactions with several other proteins which associate with DNA. The structure of sliding clamps, as resolved with the help of methods such as NMR and X-ray crystallography, is now available from many organisms [8,9,11,12,13,14,15]. Sliding clamps are symmetrical in nature and are generally arranged in a head-to-tail fashion. The stoichiometry of the subunits differs in different realms of life. For example, while they are dimeric in bacteria, they are available as a trimer in bacteriophage and eukaryotes. The job of loading of clamp onto the DNA is carried out by another set of proteins called clamp loader complexes (CLCs), which help the clamp to get positioned on the DNA in an active conformation. The clamps are responsible for managing and regulating proteins that bind to them and establish connectivity among different cellular pathways. The clamp is known to function in various channels, such as cell cycle regulation, apoptosis, DNA methylation, replication, and repair pathways, etc. These clamps, thus, along with their role in replication, are also important in various other activities to prevent genome instability.

Structural and functional aspects of the sliding clamp.

### 1.1. Genetic Organization

β clamp, encoded by the *dnaN* gene, is present universally in prokaryotes [16]. The gene locus and the gene arrangement are extensively conserved in these organisms. The arrangement includes genes for the replication initiator protein DnaA, sliding clamp, and RecF, which is involved in recombination. *dnaN* is present in between the *dnaA* and *recF* genes. Though the promoters for *dnaN* are a feature of the preceding *dnaA* region, the transcription from these promoters occurs independently [17,18]. This conservation of the gene order is likely because these proteins together carry out an essential role in the replication process. Similarly, the eukaryotic counterpart proliferating cell nuclear antigen (PCNA) is also highly conserved. PCNA and β clamp have no homology in the sequence, but their structures are superimposable.

### 1.2. Structural Organization

**Figure 1:**
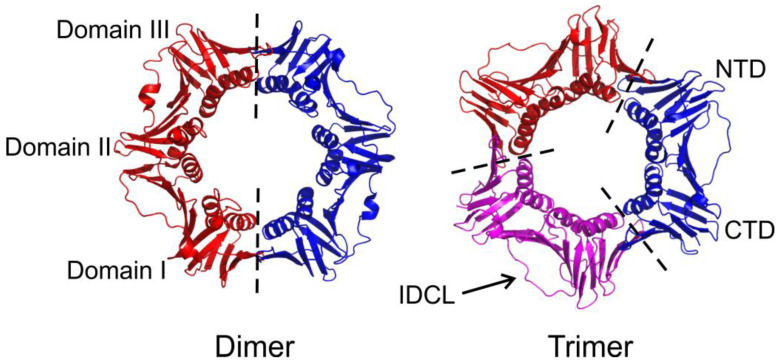
Comparison of a dimeric and a trimeric sliding clamp. The panel shows a comparison of a dimeric and a trimeric clamp. The dimeric clamp has three domains viz. domain I, domain II, and domain III, whereas a trimeric clamp has two domains viz. NTD (N-terminal domain) and CTD (C-terminal domain) per subunit. The broken line marks the subunit-subunit interface. Within a subunit, the two domains are connected through an amino acid region called the inter-domain connecting loop (IDCL). The image was generated using the *Escherichia coli* β-clamp (PDB ID: 1MMI) and human PCNA (PDB ID: 1VYM).

**Figure 2:**
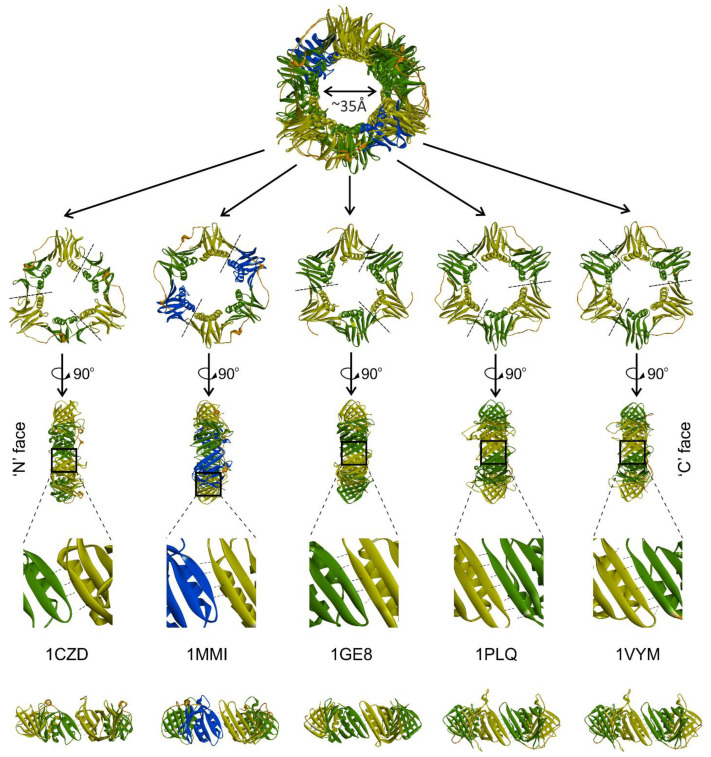
Sliding clamps from different organisms. Sliding clamp from T4 bacteriophage gp45 (1CZD), *E. coli* β clamp (1MMI), *Pyrococcus furiosus* PCNA (1GE8), *Saccharomyces cerevisiae* PCNA (1PLQ), and *Homo sapiens* PCNA (1VYM) are superimposed using PyMol. In the dimeric bacterial clamp (1MMI), three domains are colored in blue (I), green (II), and yellow (III), while in trimeric clamps, NTD is colored green and CTD, yellow; the IDLC is colored orange. The ‘N’ and ‘C’ faces of the clamps are shown. The enlarged subunit–subunit interface and hydrogen bonds are shown at the bottom of the figure.

While the sliding camps of all the domains of life share structural homology, the stoichiometry of the subunits differs [19]. The structure of the sliding clamp fulfills the utmost vital criteria viz. interactions with DNA and various other proteins. An archetypical sliding clamp has a pseudo-six-fold symmetry, and irrespective of the oligomeric state, six domains form the complete ring shape (Figure 1). These six domains are in the form of either a dimer with three domains in each subunit, as in the case of prokaryotes, or a trimer with two domains in each subunit, as found in eukaryotes and some bacteriophages [15]. The subunits arrange themselves in a head-to-tail fashion to form a complete ring. Such an assembly provides two distinct faces, namely, the N-face (amino-terminal end of the subunit) and the C-face (carboxyl terminal end of the subunit) (Figure 2) [20,21]. It has been found that the C-face acts as the major hub for the interaction with different proteins, including DNA polymerases. At the same time, the N-face is known to act as a site for post-translational modifications such as those identified in PCNA [22,23,24]. The independent domains of all the clamps are connected via the inter-domain connecting loop (IDCL), which also acts as a platform for interactions with various proteins [25].

Because of the head-to-tail arrangement of the subunits, the N-terminal domain (NTD) of one subunit interacts with the C-terminal domain (CTD) of another subunit to form the subunit-subunit interface [11,15,26]. The residues present at the interface of the subunits are very important as they establish contact between the subunits through hydrogen bonds (Figure 2) and/or intermolecular ion pair interactions, and thus play an important role in oligomerization. Indeed, reports have demonstrated that hampering these interactions affects the oligomeric nature and the overall stability of the protein [27,28,29,30]. Computational analysis shows that along with these interface residues, some conserved hydrophobic residues are equally significant in maintaining the ring shape and oligomeric nature [31]. Independent domain analysis of the T4 bacteriophage sliding clamp gp45 shows that CTD is a more stable and rigid domain, whereas NTD is more fragile and flexible. This suggests that NTD provides dynamicity to gp45, allowing the ring to open at the subunit interface, while the rigid CTD allows the protein to reform a closed trimer on DNA [26]. A dimeric ring has been suggested to be more stable than a trimeric arrangement [32,33]. However, based on experimental evidence, it has been suggested that a less stable sliding clamp could be advantageous in the replication of lagging strand as lesser stability may help in the dissociation and recycling of the clamp quickly [34]. It may also provide a functional benefit as a less stable trimer can be deployed for other moonlighting roles apart from replication.

The ring structure leads to the formation of a central cavity of ~35 Å diameter (Figure 2), which encircles the double-strand DNA (dsDNA). The structure of the PCNA and β clamp contains both α helices and β sheets. Each domain has a two-fold repeat of a βαβββ structural motif. Twelve long helices perpendicular to DNA are present in the central cavity and span the major and the minor grooves of the dsDNA. Though a few acidic residues are present, the overall charge of the helices is positive due to the presence of the Lys and Arg residues. Thus, the central cavity is lined by various Lys and Arg residues, and despite the fact that the sliding clamp is a negatively charged protein, the central cavity has a positive potential. This is essential to track the dsDNA and establish non-specific contacts between the protein and the negatively charged phosphate backbone of the dsDNA [8].

Furthermore, a few reports suggested a tilt of about 22° at the DNA axis so as to be in contact with the walls of the central cavity [11,35]. After much scrutiny over the past few years to establish the above finding, surprisingly, it was concluded that the water layer plays an important role in the interaction of the clamp with DNA, and there is no direct contact established between the sliding clamp and the positively charged residues of the dsDNA in the presence of polymerase. This ‘water-skating’, as hypothesized in an earlier report, allows for faster movement of the clamp, and the energy utilized for the same is almost negligible [36].

**Figure 3:**
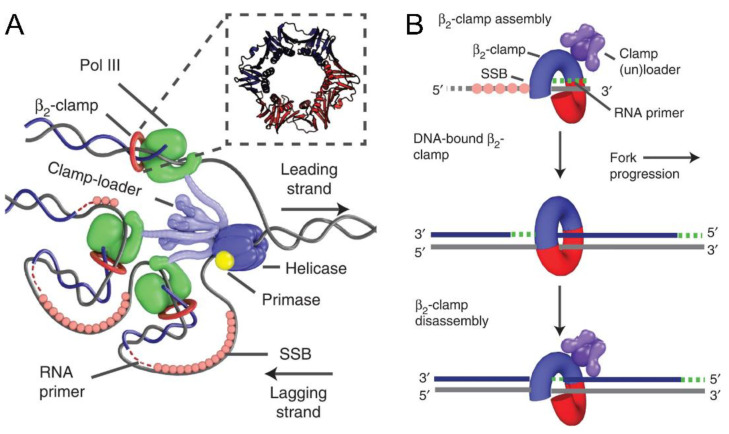
The role of the sliding clamp during replication. (**A**) The figure represents the *E. coli* replisome and the position of the clamp. The sliding clamp is loaded onto the primer-template junction by the clamp loader complex. The clamp consists of two hydrophobic pockets through which the polymerase and other proteins of the replisome interact with it. (**B**) The clamp loader complex opens the clamp, loads it onto the DNA, and is then ejected with the hydrolysis of ATP. Once the synthesis of the Okazaki fragment is completed, the clamp, along with polymerase III, is released. Reprinted/adapted from ref [42] under the terms of Creative Commons license (full terms at http://creativecommons.org/licenses/by/4.0/ (accessed on 3 November 2022)).

If present in a large number, sliding clamps may get loaded onto the DNA, though the active conformation is not achieved [37]. Although there are reports suggesting possible direct interactions between DNA and the clamp [11], the closed ring structure still needs help from another protein assembly known as the clamp loader complex (CLC) in prokaryotes and bacteriophages, and the replication factor C (RFC) in eukaryotes. These protein assemblies belong to the AAA+ ATPase family and help the clamp to load onto the DNA. The apparatus follows a simple mechanism for this procedure. When bound to ATP, the clamp loading machinery binds and opens the clamp so as to recruit it on to the primed DNA template. After the sliding clamp is positioned in an active conformation, the ejection of the clamp loader complex/RFC is followed by hydrolysis of the bound ATP (Figure 3) [38]. Based on the stability of different clamps in solution, the binding of the clamp loader complex with the clamp differs. The T4 clamp is thought to be present in an open conformation [39]; therefore, it can be easily trapped by the CLC. However, in the case of PCNA, as well as β, the clamp is considerably stable and the loader needs to open it at the subunit interface to bind to it and load it on the DNA [38,40,41]. Thus, it is impossible to achieve a perfectly active conformation of the clamp on the dsDNA without the clamp loader. Both proteins lie at the heart of the replisome, and a processive replication process is not possible without these two.

### 1.3. Sliding Clamps as Processivity Factors

Since its discovery, the basic and foremost function of a sliding clamp in all domains of life is to complement the polymerase activity by enhancing its processivity, which is required by the cell to boost its replication rate and thereby improve the cell division process. Reports suggest that the rate of incorporation of nucleotides increases from tens to thousands of nucleotides per second when the sliding clamp comes into the picture, along with the polymerase [43]. DNAP binds to the hydrophobic pocket present at the C-terminus of the clamp and the DNA tilts at an angle of ~22° passes through the center of the ring [11].

When these DNA-encircling proteins were discovered, it was assumed that cells evolved such protein machinery only for ‘holding’ the polymerase and preventing it from ‘falling off’; hence the name processivity clamps [44]. However, later, various reports suggested plentiful proteins other than the polymerases, which interact with these clamps [45]. In the T4 phage, for example, it has been shown that the gp45 (T4 phage sliding clamp), besides being involved in DNA replication, is also involved in enhancing transcription of late genes by interacting with transcription factors gp33 and gp55 [46]. However, several studies have reported the presence of polymerases such as the phi29 DNA polymerase and T7 DNA polymerase, which are highly processive and do not require aid from the clamps [47]. Therefore, one can argue that clamps did not evolve just for the purpose of speeding up and making the process of replication processive, but they may have other important functions too.

### 1.4. Beyond the Obvious: Functions Other Than Processivity

Many different proteins involved in DNA replication, repair, and other cellular processes have been identified so far, which require establishing contact with DNA. The sliding clamp acts as the chief conductor for the orchestra of such proteins. The binding sites for the ligands are the IDCLs and the underlying hydrophobic pockets. The clamp binding proteins usually contain a consensus sequence motif through which they can access the clamp. In bacteria, β clamp-binding proteins have a conserved pentapeptide motif QL(S/D)LF at their C-terminus [48]. It is, however, possible that this consensus sequence accepts much more variability, and therefore, identification of the clamp binding motifs (CBM) in prokaryotes is somewhat difficult. PCNA-interacting proteins (PIPs) preferably have a stricter sequence motif Qxx[I/L]xxFF with much less variability [49]. However, there are reports available that suggest that PIPs are not the only motifs, and that there are other PIP-like motifs that essentially serve the same purpose [50].

### 1.5. Interactions with Other Members of the Replication Machinery

Apart from DNAP, the sliding clamp is known to interact with other participants of the replication process. In bacteria, the initiation of DNA replication is dependent on DnaA. Active DnaA (DnaA-ATP) is loaded on the *oriC* and is further responsible for the recruitment of helicase. Subsequently, the other events of the replication unfold. The initiation step has to occur only once per replication cycle, and in order to ensure this, several modes of regulation are present. One such mechanism is the hydrolysis of the bound ATP to yield an ADP-bound inactive form of DnaA after the synthesis of DNA begins. This method of regulatory inactivation of DnaA (RIDA) includes a DnaA-like protein Hda and β clamp [51,52]. Hda stimulates the ATP hydrolysis of DnaA by interacting directly with it. This interaction requires the sliding clamp as a mediator, which maintains its contact with had, as well as with the location on DNA where the replisome is present [53]. Similar observations were made while studying RIDA in *Caulobacter crescentus*, which hinted that clamp is essential to direct Hda at the site occupied by the replisome [54]. A similar mechanism is also employed in eukaryotes to prevent re-initiation of replication. The pre-replication complex (pre-RC) includes a protein called Cdt1, which has a distinctive PIP-motif named as PIP-degron with a higher affinity for the PCNA. The favoured tight association of this protein with PCNA marks it for polyubiquitylation and, in turn, causes its degradation [55]. Thus, Cdt1 can no longer cause re-licensing of the origin.

During the synthesis of the lagging strand, when Okazaki fragment maturation is done by DNA polymerase I with the help of the DNA ligase to seal the gaps, the sliding clamp is present on-site to play a central role. In *E. coli*, the sliding clamp is known to increase the efficiency of ligation through two methods. Firstly, it makes direct contact with the DNA ligase, and secondly, it slows down the nick translation process and enhances the 5′ exonuclease activity of DNA polymerase I. Thus, early ligation is favoured by the sliding clamp [56]. Such interaction can also be seen between the *Helicobacter pylori* sliding clamp and ligase [57]. However, one report from *Mycobacterium tuberculosis* suggests that no such direct interaction between these two proteins exists [58]. Studies carried out in eukaryotes have also confirmed the role of PCNA as a mediator to which all the proteins involved in Okazaki fragment maturation come and bind cyclically. For example, proteins such as FEN 1, polymerase δ, and ligase are included in the interactome of PCNA in *S. cerevisiae*, as well as mammalian model systems. The conformational changes in these binding partners drive the difference in the specificity of PCNA towards them [59].

In the eukaryotic replication system, Pol δ has the responsibility to carry out the synthesis of lagging strand with the help of PCNA. After the strand displacement by Pol δ-PCNA, the 5′ flap is removed by FEN-I and the Okazaki fragments generated are ligated together by LigI. Depending on the number of functional binding sites available on the trimeric clamp, all three proteins listed above can bind either cyclically [60] or simultaneously [61], as has been shown for yeast and humans, respectively. After finishing its job successfully, Pol δ dissociates from the DNA but remains bound to PCNA, and thus, the number of FEN-I molecules associating with PCNA is limited. It was also observed that LigI interacts with Pol δ, and a stable Pol δ-PCNA-FEN-I-LigI complex is formed on the DNA. Once the 5′ flap is removed, Pol δ and FEN-I dissociate from PCNA. Now, LigI can directly associate with the clamp and the nick can be effectively sealed. Therefore, by allowing these three proteins to bind, PCNA manages the process of Okazaki fragment synthesis and maturation [61].

### 1.6. Transcription Activation and the Clamp

**Figure 4:**
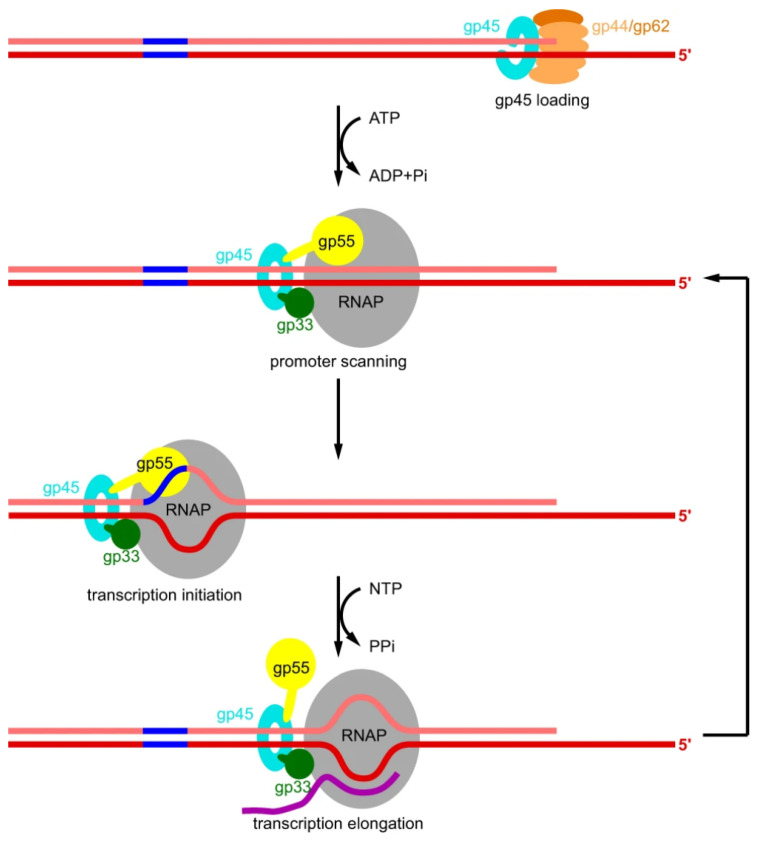
gp45 mediated transcription activation. After the gp45 is loaded onto the DNA, gp55 and gp33 mediate the binding of gp45 with the RNAP. The three proteins, along with the RNAP, start the promoter scanning process along the DNA, and initiate transcription upon interaction with a promoter. Gray, RNAP; light orange, gp44; dark orange, gp62; cyan, gp45; yellow, gp55; dark green, gp33; salmon, nontemplate-strand DNA; red, template-strand DNA; blue, -10-like element; magenta, RNA. Reprinted from ref [65] under the terms of the Creative Commons CC BY license (full terms at http://creativecommons.org/licenses/by/4.0/ (accessed on 3 November 2022)).

A unique role of the T4 bacteriophage sliding clamp gp45 was established in the late 1990s. It was noted that apart from replication, the T4 gp45 has a very crucial role in activating the transcription of late genes, along with the T4 sigma factor gp55 and a co-activator protein gp33 [62,63]. gp55 and gp33 interact with gp45 through the LDFL motif present at their C-termini [64]. While gp55 is capable of carrying out basal transcription from the T4 late promoter, the binding of gp33 suppresses this transcription. However, interaction with gp45 with the RNA polymerase holoenzyme (RNA polymerase + gp55 + gp33) enhances the transcription several folds [64]. Thus, gp45, the sliding clamp of T4 phage, moonlights as a transcription activator, and, in a manner, couples transcription with phage DNA replication. A model by Geiduschek and Kassavetis explains the gp45-mediated transcription initiation [46]. To initiate the transcription, the RNA polymerase needs to locate the promoter sequences. Sliding clamp gp45 acts as a DNA-tracking protein in this case. Due to the ATP hydrolysis, T4 clamp loader complex (composed of gp44 and gp62 proteins) and gp45 dissociate from each other and the latter stays on the DNA. This orphaned clamp now meets gp33 and gp55 while sliding on the DNA, and the three move together and search for the promoter [46]. The recent cryo-EM structure elaborates the entire process in a concordant manner with the proposed model. It was observed that gp55 causes a blockage in the RNA exit channel and may thus stop the further extension process. Therefore, this roadblock has to be removed. In comparison, gp33 and gp45 do not cause any hindrance and thus remain attached to the RNA polymerase. The presence of a sliding clamp binding motif on gp55 enables it to stay attached to gp45, even after it is displaced from RNA polymerase. Thus, soon after transcription termination, the promoter scanning process is resumed (Figure 4) [65].

An in-depth mutational analysis of gp45 showed the significance of the two domains of the gp45 monomer in the protein’s stability and activity in terms of both transcription and DNA replication. Interestingly, the data suggested that certain mutations, such as Q125A or K164A, located in the C-terminal domain of trimeric gp45, allowed the protein to support only DNA replication and not late promoter transcription. Thus, this study indicated that the C-terminal domain of the trimeric gp45 is likely involved in the late promoter transcription activation [27].

### 1.7. Role in Maintenance of Genome Integrity

**Figure 5:**
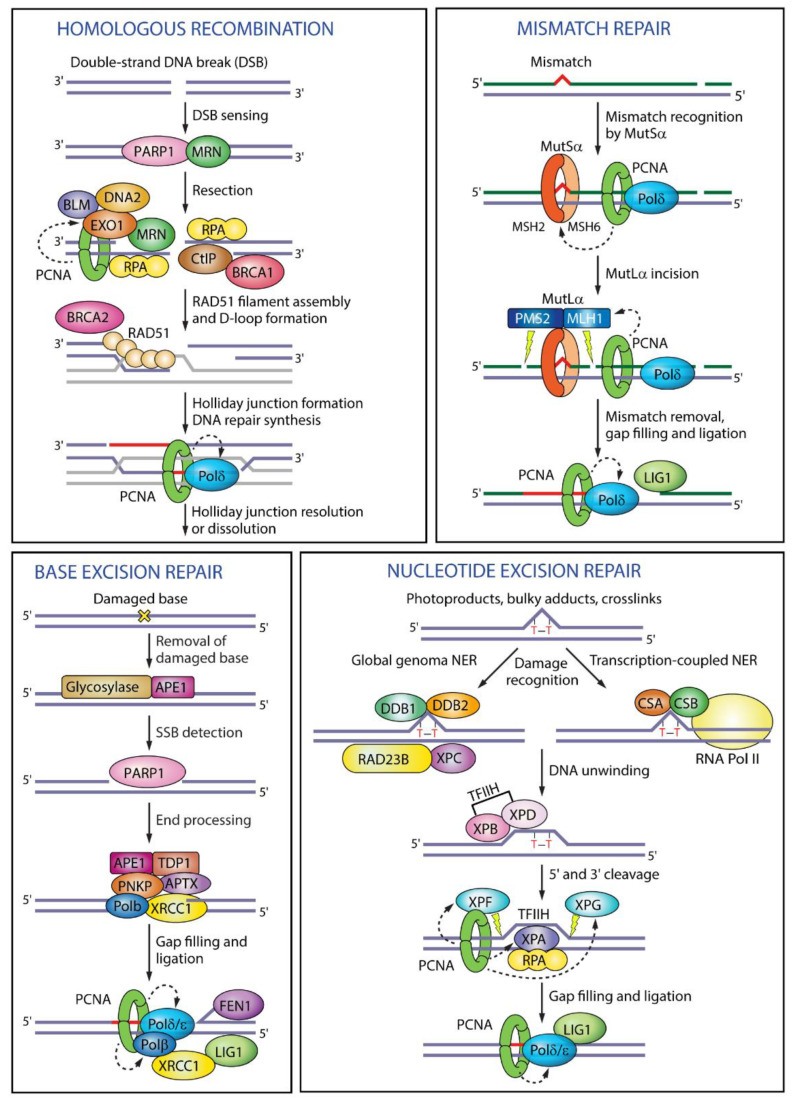
Overview of PCNA in DNA repair mechanisms. Reprinted from ref [66] under the terms and conditions of the Creative Commons Attribution (CC BY) license (full terms at http://creativecommons.org/licenses/by/4.0/ (accessed on 3 November 2022)).

Fidelity, along with processivity, is a necessary characteristic of cellular replication. There are many factors responsible for causing serious DNA damage. If not rectified and controlled, these damages can lead to genome instability, thereby causing chromosomal aberrations and life-threatening diseases. According to the nature of DNA damage, multiple DNA repair mechanisms have been identified. They include mismatch repair (MMR), break induced replication (BIR), base excision repair (BER), and translesion synthesis (TLS), among others. Sliding clamp is involved in many of these processes too (Figure 5).

Mismatch repair is one of the post-replicative damage repair pathways, which exist in almost all organisms with a few exceptions. MutS, MutL, and MutH and their eukaryotic homologs, namely MSH and MLH, are involved in rectifying the mistakes caused by DNA polymerase. The C-terminal of MutL has the endonuclease activity and also harbours the β clamp binding motif [67]. MutH is not present in all prokaryotes, and the endonuclease domain of MutL compensates for its absence. The sliding clamp is also known to recruit MutS, which is responsible for identifying the mismatches. A previous report by Simmons et al. confirmed that the β clamp plays a critical role in stabilizing and recruiting MutS at mismatched sites. Additionally, when a temperature-sensitive allele containing a G73R mutation was examined, it failed to aid in the MMR pathway. The increase in mutation frequencies in these allele was attributed to the failure of MMR instead of any other repair pathway [68]. Similarly, in eukaryotes, PCNA is known to interact with MutS and MutL homologs to serve in the MMR. The endonuclease domain of the MutL homolog is said to be activated via PCNA [69].

Interestingly, in the phylum actinobacteria and some archaea, although the genes related to the mismatch repair pathway are absent, the mutation rate is similar to that of an organism with MMR. This finding hints at the presence of some other methods by which these mutations are corrected [70]. Reports are available that suggest that some organisms, such as *Mycobacterium* species, *Corynebacterium glutamicum* etc., possess a protein, NucS, that has mismatch-specific endonuclease activity (EndoMS), and, hence, it is called NucS/EndoMS. In the absence of this protein, the rate of mismatches increases spontaneously. The presence of the β-clamp binding motif and crosslinking studies suggested interaction of the sliding clamp with EndoMS in *C. glutamicum*. This direct interaction is responsible for lower mutation rates. It was also observed that tethering of the sliding clamp with EndoMS leads to increased cleavage by the protein. It was further hypothesized that the positioning of the EndoMS by the clamp at the mismatch site might play a role in detecting errors. Additionally, the clamp might allosterically activate the enzymatic activity of the endonuclease [71]. Thus, although the proteins involved in the mismatch repair might differ in different organisms, the sliding clamp nevertheless plays a central role in guiding and correctly recruiting these proteins.

Another kind of DNA repair pathway is the base excision repair (BER). As the name suggests, the chemically altered or damaged base is identified and excised by DNA glycosylases/endonuclease via this pathway. Further, the thus-generated abasic site is repaired by other proteins involved in the process. BER is very efficient in the removal of these bases and is, therefore, important for genome maintenance. It is also known to protect against many diseases, including Huntington’s disease, as well as ageing [72].

In bacteria, Nei proteins are known to remove oxidized bases from DNA substrates. In *M. tuberculosis,* it has been reported that Nei2, with its peculiar clamp binding motif, interacts with subsites I and II, which are present near the interface of the dimeric clamp. This interaction enhances the Nei2 activity by many folds [73]. It was also observed that clamp interaction with another endonuclease, XthA, is of considerable importance for mycobacterial BER [74].

Similarly, in mammalian systems, there are around 11 DNA glycosylases present, out of which Neil, though it lacks a PIP motif, surprisingly binds to a disordered C-terminal region of PCNA. This protein is also known to be stimulated by interaction with PCNA at IDCLs. Through AFM analysis, it was observed that with Neil1, PCNA is not found in its regular trimeric form, thus suggesting an entirely different kind of regulation and switching from replication to repair [75]. Another DNA glycosylase involved in mammalian BER is a type of adenine glycosylase, known as mammalian MutY homologue (MUTYH). It binds via the regular PIP box and removes the adenine-paired opposite to 8-oxo-Guanine generated because of ROS. PCNA provides a fixed direction to MUTYH for the removal of the mispaired adenine [76]. Thus, both bacterial and eukaryotic clamps are involved in the genome maintenance.

### 1.8. Translesion Synthesis and the Clamp

Occasionally, when the template to be replicated is severely damaged, and the DNA repair methods discussed above fail to mitigate the damage, it leads to replication stalling. The cells, in such a scenario, need a way out to continue the replication of the genetic material. The regular polymerase is of no help in these cases. At such times, cells respond via two strategies: translesion DNA synthesis and non-mutagenic damage avoidance [77]. A separate Y-family, low-fidelity polymerase is engaged so as to maintain the replication past these lesions. Such synthesis is termed translesion DNA synthesis (TLS) [78]. The process of TLS is prioritized over the damage avoidance pathways as the low-fidelity polymerases ensure genetic variability [77]. The Y-family polymerases include polymerase IV and V in prokaryotes, and Polŋ and Rev1 in all eukaryotes, along with Pol ɩ (vertebrates) and Pol κ [78].

When a replication block occurs, and pol III cannot carry out the synthesis any further, Pol V binds at the damaged site. With the help of its interaction with the RecA and the β clamp, pol V carries out the process of TLS [79]. Crystallographic evidence suggests that in *E. coli*, pol IV is also known to enter the replisome under such circumstances; nevertheless, both polymerases share the same binding site on the β clamp [80]. Once pol IV, the error-prone polymerase, replicates the damaged part, pol III takes over for further synthesis [81]. In the eukaryotes, many post-translational modifications regulate interactions of PCNA and thus assist in TLS. The process of TLS is said to be in control of monoubiquitination. It is proposed that the differential affinity of various Y-family polymerases towards PCNA determine which of them is to be recruited on the primer terminus [82]. The modified PCNA thus switches from the high-fidelity polymerases to the low-fidelity ones. The Mms-Ubc13-Rad5 complex can further polyubiqutinylate PCNA and cause template switching, thus leading to error-free lesion bypass. In a similar manner, sumoylation of PCNA also prevents replication fork collapse [83].

All the repair pathways and the proteins involved in them are absolutely essential for genome maintenance. Loss or mutation in even one of them can cause havoc in a replicating cell. The replication factor C-like complex, which acts as a clamp loader, is one such critical protein. Its major subunit, Elg1 (ATAD5 in mammals), unloads PCNA from DNA and is the one that ensures genomic integrity [84]. The absence of Elg1/ATAD5 leads to the abnormal accumulation of PCNA on the DNA. Studies have concluded that indeed the fundamental cause for the loss of genome stability is the problematic and unusual retention of PCNA [85,86].

The exchange of polymerases involved in elongation and repair or ‘Polymerase switching’, is a critical process to ensure a correct and uninterrupted replication process. The sliding clamp acts as a mediator between the polymerases. The polymerase switching and the role of clamp in this process were first discovered in the T4 bacteriophage. It was advocated that in the T4 phage, there can be either a momentary attachment of the arriving polymerase with the inter-domain region of the clamp before exchange or a direct-displacement of the already attached polymerase [87]. A well-known ‘tool-belt’ model suggests that two polymerases can simultaneously bind the clamp while their interaction affinity drives the switch [88]. However, some reports contradict this model and assume that only one polymerase can attach at a time to the sliding clamp. In 2013, Rego et al. proposed that the replicative polymerase pol III of *E. coli* establishes contact with the sliding clamp at two sites. The exonuclease domain is responsible for this extra but weaker interaction of pol III with the clamp. When the replication gets stalled and the damage is not repairable, pol IV comes to the rescue. The increased concentration of this polymerase and the weaker interaction at the exonuclease domain are responsible for the removal of the pol III from the damage site [81]. Thus, the interaction points between the polymerase and the clamp play a pivotal role in regulating TLS.

In *S. cerevisiae*, it was shown that the PIP motif was crucial in polymerase exchange. Furthermore, the stalling of polymerase δ and monoubiquitination of PCNA were a pre-requisite of polymerase η binding. The modifications in PCNA may be essential for attracting and providing a certain favourable conformation to pol η. Once the damage is repaired, the regular polymerase is available again, but only after PCNA undergoes deubiquitination [89]. Thus, both the repair and replication can go hand in hand uninterruptedly, with the sliding clamp being their bridge.

### 1.9. Transposon Interaction with β Clamp

In 2009, it was shown that transposon insertion in replicating DNA occurs through β clamp. Experimental evidence suggests that β clamp interacts physically and functionally with the Tn7 transposon-encoded protein TnsE and dictates their selective introduction and activation. Interaction with TnsE (foreign protein) does not hamper the regular physiological roles of the β clamp inside the cell [90]. Many other insertional sequences (ISs) were later shown to functionally interact with the β clamp [91]. This suggested that transposition and replication are linked and occur in a well-coordinated manner.

### 1.10. Other Moonlighting Roles of PCNA

The moonlighting functions discovered till date for the bacterial sliding clamp are somewhat lesser known than its eukaryotic counterpart. The increased complexity of eukaryotes might be one of the many reasons for the diverse roles of PCNA. Apart from the position in replication and repair, this versatile protein can perform several other functions. The fact that many organisms do not depend on the clamp for increasing the processivity leads to the proposal that the clamps did not naturally evolve just for the sole purpose of providing processivity. Instead, the polymerases evolved themselves to depend on clamps to fulfil other important functions. Thus, the dependency on the clamps is the ‘cause’, and the ‘effect’ is the accumulation of clamps on the newly replicated DNA. This hypothesis paves the way for understanding different activities occurring on daughter DNA duplexes ‘marked’ by PCNA [44].

Nucleosome assembly is one such event where the localization of PCNA on new DNA further brings in proteins such as the chromatin assembly factor (CAF-1), thereby causing an assembly of H3 and H4 histones [92]. PCNA is also thought to mediate the methylation of newly replicated DNA via DNA cytosine methyltransferase (Dnmt 1), the enzyme responsible for the CpG methylation. Dnmt 1 has a PCNA-binding motif, and therefore, has a greater affinity for PCNA-bound DNA than free DNA. This leads to an increased efficacy of selective and specific methylation on hemimethylated daughter DNA [93].

It has now been established that PCNA has a considerable number of partners to associate with (Table 1), and the extent of the binding efficiency determines the role to be played by this extraordinary mediator. It has a reputation to usher many proteins involved in cell cycle regulation, cell signaling, and apoptotic pathways. For example, UV-irradiated ING 1, a tumour suppressor protein, binds tightly to PCNA leading to the commencement of apoptosis. There are also many anti-apoptotic proteins, such as Gadd45 family, which have a PIP-box. The binding of these proteins to PCNA causes their inactivation and thereby inhibits apoptosis. PCNA is also involved in regulating the stability of p53, a tumour suppressor protein [25].

Owing to the large number of proteins that can interact with PCNA, as well as its role in cell proliferation, the role of PCNA in cancer is of great interest. It is now widely known that the majority of PCNA functions are controlled via post-translational modifications. A Tyr residue present at the 211 position is known to get phosphorylated by EGFR and c-ABL tyrosine kinase. This phosphorylation helps in promoting cell proliferation in cancerous cells, and blocking the phosphorylation of this residue, in turn, leads to tumour suppression [24].

Cancer cells are known to be immortal. To achieve this immortality, they have to stop the telomerase shortening process. This is usually accomplished by two methods: either by extension with the help of Telomerase, a reverse transcriptase, or through the alternative lengthening of the telomeres (ALT) pathway. In contrast to the cancer cells containing telomerases, cells undergoing the ALT pathway are more prone to replicative stress, dsDNA breaks (DSBs) and replication blocks. In order to avoid various chromosomal aberrations and rearrangements, this stress should be mitigated. PCNA is known to mediate in this matter. It interacts with RTEL1, which resolves the G4 structures formed due to repetitive G-rich sequences of telomeres. Apart from this, it also recruits the pol δ complex at telomere synthesis sites. Thus, because of such strong roles in cancer progression, PCNA is considered as an effective target for the disease treatment [94,95].

In neutrophils, PCNA has been found to be associated with various procaspases in the cytosol to prevent their activation, thereby preventing apoptosis and governing the neutrophil survival. Various post-translational modifications could be responsible for this association [96]. Furthermore, one of the two PCNA-interacting motifs, APIM, found in eukaryotes has been shown to be conserved in the proteins of PI and MAPK pathway in yeast and humans, which suggests that PCNA acts as a platform for various protein interactions in the cellular stress response [97].

### 1.11. Unusual Processivity Factors

#### 1.11.1. Viral Clamps

Computational analysis of viral replisomes suggested that a few viruses lack clamp loader complexes [98]. Sliding clamps of such viruses are high in positive charges and directly bind to the dsDNA. These clamps are not ring-shaped and they stay attached to their respective DNA polymerases either as monomers (UL42) or dimers (UL44). The proposed structure of the Herpes simplex virus clamp UL42 shows that DNA polymerase binds to the outer β-sheet and the clamp interacts with DNA through a positively charged α-helix [98,99]. The Human Cytomegalovirus clamp UL44 is a C-shaped dimeric clamp that looks like a hybrid of PCNA and UL42 [100]. The computational analysis shows that viral clamps can be categorized as either cellular (β clamp or PCNA) or viral (gp45, Ul42, UL44, and BMRF1) homologues [98].

#### 1.11.2. 9-1-1 Clamp

*Schizosaccharomyces pombe*, *S. cerevisiae* and *H. sapiens* have a unique heterotrimeric clamp-like protein called a 9-1-1 clamp because each subunit is encoded by RAD9 (396 aa), RAD1 (286 aa), and HUS1 (283 aa) [101]. Although PCNA and 9-1-1 clamps have insignificant sequence similarity, they are structural homologues, and the 9-1-1 clamp has also been referred to as ‘PCNA’s specialized cousin’ in the literature [102]. The 9-1-1 clamp has also been found to hold significance in checkpoint control; hence the name, checkpoint clamp [103]. It interacts with Chk 1, which is a protein kinase that regulates S-phase progression, G2/M arrest, and the replication fork stabilization [104]. Furthermore, 9-1-1 clamp has also been shown to have a role in DNA repair [103]. Similarly, *Sulfolobus solfataricus*, a hyperthermophilic archeon, is known to have PCNA composed of three distinct proteins, which interact together to form a heterotrimer and participate in DNA replication [105].

#### 1.11.3. Thioredoxin as the T7 Clamp

Bacteriophage T7 replisome machinery does not have a sliding clamp. Interestingly, its DNA polymerase uses thioredoxin, a small redox protein, to enhance its processivity. Therefore, to stay tethered to the replication fork, the T7 DNA polymerase binds to helicase [106,107]. During DNA damage, unlike other replisomes, the T7 DNA Polymerase-Helicase complex remains associated with the DNA and executes replication through lesion without getting dissociated from DNA [108]. Polymerase switching in the T7 replisome occurs rather easily; the incoming polymerase passively binds the helicase and replaces the synthesizing polymerase without affecting replication processivity and fidelity [109,110]. This is somewhat similar to T4 replisome dynamic processivity.

### 1.12. Utilizing the Potential of Clamps: Development as Therapeutic Targets

A vast repertoire of proteins has been identified till date, whose functions are modulated via the sliding clamps. Not only in eukaryotes but also in prokaryotes, this interactome is being identified continuously. The ability of the clamps to affect and regulate many different cellular processes makes them a strong candidate for the development of new therapeutic targets. The hydrophobic cleft, at which all of the clamp-interacting proteins bind, via their clamp-binding motif, can be a good target for the same. Specific small molecule inhibitors, peptides, natural products, non-steroidal inflammatory drugs, etc. have been developed that strongly bind to the hydrophobic clefts of the clamps to inhibit replication and repair pathways [111,112]. Various studies on bacterial-encoded toxins also encourage the identification of new drug targets. For example, in SocAB, a toxin-antitoxin system found in *C. crescentus*, the toxin SocB inhibits the elongation step of DNA replication by binding to the sliding clamp via the usual clamp-binding motif. The binding of SocB takes place in the hydrophobic cleft of the clamp, a site shared by many other proteins. Therefore, barring the elongation step, various other interactions could also be inhibited because of this SocB-DnaN association. Small molecule therapeutics could be designed based on this knowledge, making the sliding clamp a crucial target for disrupting vital functions in pathogenic bacteria [113].

In a recent report, it was shown that bacterial growth is inhibited by low concentrations of cell-penetrating peptides containing PCNA binding motif [114]. Certain inhibitors containing tetrahydro carbazole moieties have also been designed, which mimic the binding between linear motifs from polymerases and *E. coli* sliding clamps [115]. RU7, which is one of the first inhibitors to be developed, is known to inhibit polymerase II, III, and IV [111]. The sliding clamp has also been considered as a potential drug target against *M. tuberculosis*. Grieslimycin, a derivative of streptomycin, was found to be active against the bacterium by inhibiting its sliding clamp and thereby replication [116]. Similar inhibition of replication was seen by the use of several non-steroidal anti-inflammatory drugs (NSAID) in *E. coli* [111].

Since PCNA is overexpressed in rapidly dividing cancer cells, it acts as a potential therapeutic target against cancerous cells. PCNA has been developed as a drug target by the formulation of peptides, which either intervene between different associating proteins and PCNA or alter the modifications of the PCNA. One well-known protein, p21, is known to interact with PCNA very strongly. Peptides derived from the PIP motif of p21 associate with the IDCLs of PCNA and, therefore, make the site unavailable for several other interacting partners [117]. Prevention of post-translational modifications of PCNA by inhibitory peptides is also one of the many ways through which processes, such as cell growth, etc. can be modulated. Additionally, small molecules targeting PCNA trimerization have also been designed and have been shown to be effective against tumour growth [118]. Studying how some diseases progress and how certain pathogenic bacterial proteins interact with the sliding clamps gives insight into the mechanism by which the basic cellular pathways can be hampered. This would help in the development of novel anti-microbial and anti-cancer drugs [24,117].

## 2. Conclusions

**Figure 6:**
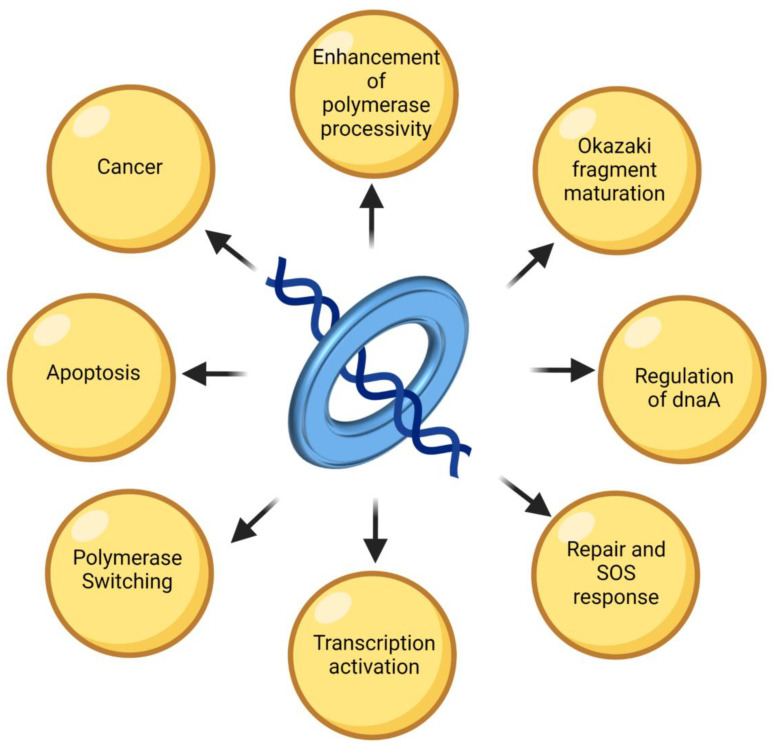
The many roles of a sliding clamp. The image shows the many physiological processes in which the sliding clamp is involved in bacteriophages, prokaryotes, and eukaryotes. The image was created with www.biorender.com (accessed on 28 September 2022).

The DNA polymerase executes DNA replication with high fidelity and processivity. The processivity is contributed by the sliding clamp protein that binds to DNA polymerase during DNA synthesis. However, the sliding clamps have come a long way from being mere processivity factors to becoming a ‘Jack of all trades’. These polymaths, with wide-ranging communication abilities, lie at the heart of several critical pathways in a cell. The presence of a conserved binding motif in all interacting partners allows them to associate with the clamp and carry out their necessary functions in a coordinated manner. The ‘tool-belt’ model can explain how this heavy traffic is regulated. Moreover, the presence of nearly the same consensus sequence in all the interacting proteins sheds some light on how evolution has resulted in the same protein mediating different pathways, and thus there is connectivity between them. The presence of similar secondary clamp-binding motifs in some proteins further fine-tunes their regulation. The clamps also mark the newly replicated DNA for nucleosome assembly and chromatin modelling. Further structural studies on clamps of different organisms can elaborate on the structural-functional relationships and how the regulation of various proteins involved in replication and repair is refined. Nevertheless, the existing knowledge on both prokaryotic and eukaryotic sliding clamps is vast and establishes their role not only as proteins that hold the polymerase on the DNA, but also as proteins which help in maintaining error-free replication, DNA methylation, cell-cycle progression, apoptosis, etc. (Figure 6). Thus, the significant roles played by sliding clamps in replication, genome maintenance, etc. also make them an attractive target for developing potent antibiotics or anti-cancerous agents.

## Figures and Tables

**Table 1:** Sliding clamp-binding proteins. Table shows the list of various cellular processes and the proteins that interact with the sliding clamp during these processes.

Process	Proteins Interacting with Eukaryotic PCNA	Proteins Interacting with Bacterial Sliding Clamp
DNA replication	DNA Polymerase δ, DNA Polymerase ε, Replication factor (RfC1, Rfc3, Rfc4), Flap endonuclease (FEN-1), DNA ligase 1, DNA topoisomerase 1, Cdt1, Topo Iiα	DNA Polymerase III, DnaA, Clamp loader complex, DNA Polymerase I, DNA ligase
DNA repair	DNA mismatch repair protein Msh3, Msh6, Mlh1, Exonuclease 1, UNG2, MPG, PARP-1, APE1, APE2, XRCC1, WRN, BLM, RECQ5, NTH1, hMYH, XPG	MutL, MutS, NucS, Nei2, XthA, DNA Polymerase IV, DNA Polymerase V
DNA damage	DNA Polymerase η, DNA Polymerase ζ, DNA Polymerase λ, DNA Polymerase κ, DNA repair protein REV1, E3 ubiquitin-protein ligase RAD18 and Rad5	
Chromatin Assembly	WSTF, DNMT1, HDAC1, p300, CAF-1	_
Cell cycle control	p21 (C1P1/WAF1), P27 (KIP2), Cyclin D1, MCL1, P15 (PAF), CDK2, ING1b, Gadd45, MyD118, CR6, P53, MDM2	DnaA, Hda
Sister chromatid cohesion	ESCO1, ESCO2, Chl1, Ctf18	_
